# Genetic Variations of *MSTN* and *Callipyge* in Tibetan Sheep: Implications for Early Growth Traits

**DOI:** 10.3390/genes15070921

**Published:** 2024-07-15

**Authors:** Kai Zhao, Xue Li, Dehui Liu, Lei Wang, Quanbang Pei, Buying Han, Zian Zhang, Dehong Tian, Song Wang, Jincai Zhao, Bin Huang, Fuqiang Zhang

**Affiliations:** 1 Key Laboratory of Adaptation and Evolution of Plateau Biota, Qinghai Provincial Key Laboratory of Animal Ecological Genomics, Northwest Institute of Plateau Biology, Chinese Academy of Sciences, Xining 810008, China; lixue@nwipb.cas.cn (X.L.); liudehui@nwipb.cas.cn (D.L.); hanbuying@nwipb.cas.cn (B.H.); tiandehong@nwipb.cas.cn (D.T.); wangsong@nwipb.cas.cn (S.W.); 2 University of Chinese Academy of Sciences, Beijing 100049, China; 3 Qinghai Sheep Breeding and Promotion Service Center, Gangcha 812300, China; wanglei1911@163.com (L.W.); peiquanbang@163.com (Q.P.); 13997124933@163.com (Z.Z.); lsj09099@163.com (J.Z.); 13086271482@163.com (B.H.); 18197156073@163.com (F.Z.)

**Keywords:** *MSTN*, *Callipyge*, SNP, growth traits, Tibetan sheep

## Abstract

**Simple Summary:**

Tibetan sheep are integral to the ecosystems and livelihoods of the Tibetan Plateau; however, their breeding practices limit their production and growth. Candidate genes myostatin (*MSTN*)and *Callipyge* have been recognized as important for improving growth traits in livestock. This research examined the polymorphic loci of these genes in Tibetan sheep and their association with growth traits. The results indicated that SNP loci of *MSTN* could potentially be utilized as a molecular indicator for early growth traits in Tibetan sheep.

**Abstract:**

Tibetan sheep are vital to the ecosystem and livelihood of the Tibetan Plateau; however, traditional breeding methods limit their production and growth. Modern molecular breeding techniques are required to improve these traits. This study identified a single nucleotide polymorphism (SNP) in myostatin (*MSTN*) and *Callipyge* in Tibetan sheep. The findings indicated notable associations between *MSTN* genotypes and growth traits including birth weight (BW), body length (BL), chest width (ChW), and chest circumference (ChC), as well as a particularly strong association with cannon circumference (CaC) at 2 months of age. Conversely, *Callipyge* polymorphisms did not have a significant impact on Tibetan sheep. Moreover, the analyses revealed a significant association between sex and BW or hip width (HW) at 2 months of age and ChW, ChC, and CaC at 4 months of age. Furthermore, the study’s results suggested that the genotype of *MSTN* as a GA was associated with a notable sex effect on BW, while the genotype of *Callipyge* (CC) showed a significant impact of sex on CaC at 2 months of age. These results indicated that the SNP of *MSTN* could potentially serve as a molecular marker for early growth traits in Tibetan sheep.

## 1. Introduction

The Qinghai–Tibetan Plateau is distinguished by its distinctive ecosystem and extreme environmental factors, including high altitude, low temperature, and intense ultraviolet radiation [1]. Tibetan sheep (*Ovis aries*) hold a prominent ecological position as the predominant and extensively distributed livestock species on the Tibetan Plateau and serve as a vital resource for the indigenous population [2]. Additionally, they play a crucial role in supporting the livelihoods of the Tibetan Plateau inhabitants by offering a dependable source of meat, wool, and leather [3]. The morphology of Tibetan sheep is characterized by a rectangular body shape, with an average body height of 68.19 cm for rams and 65.11 cm for ewes, and body lengths of 76.56 cm and 65.79 cm, respectively. The predominant color of their hair is white, with variegated patterns on the head and limbs [4]. In terms of growth performance, Tibetan sheep exhibit lower weights compared to high-quality meat sheep breeds, with adult rams weighing around 51.0 kg and ewes around 43.6 kg [5]. In contrast, Hu sheep have adult weights of 53 kg for ewes and 85 kg for rams [6], while Dubo sheep reach weights of approximately 120 kg and 85 kg for adult rams and ewes [7]. Furthermore, Tibetan sheep, being a traditional meat sheep breed, exhibit problems such as constrained productivity, sluggish growth, and diminished slaughter rates attributed to the absence of sophisticated breeding techniques. Numerous research studies have identified a wide array of candidate genes implicated in the control of metabolism and modulation of growth rates in domestic animals. [8]. Variations in these genes have been extensively employed as molecular markers to facilitate breeding and enhance productivity [9]. However, studies on Tibetan sheep are limited.

*Myostatin* (*MSTN*), also known as *GDF-8*, is a growth factor belonging to the TGF-β superfamily, located on the long arm of chromosome 2 in sheep. It is composed of three exons and two introns [10]. Predominantly found in muscle tissues, it plays a critical role in the control of development and growth through its inhibition of cell cycle progression [11]. Extensive research has demonstrated that the suppression of *MSTN* expression leads to increased meat production in animals, commonly called double-muscle animals [12,13]. A notable augmentation (2–3-fold) in skeletal muscle mass has been documented in *MSTN*-null mice [14]. Multiple investigations have recognized *MSTN* as a pivotal candidate gene that impacts productivity, growth, and performance in diverse domestic animal species, including pigs, sheep, and chickens [15,16,17]. Specifically, investigations have demonstrated that *MSTN*-KO sheep exhibit a propensity for accelerated growth compared with wild-type (WT) sheep [18]. Additionally, studies have demonstrated that mutations in *MSTN* in sheep led to notable gains in body weight and muscle mass [19].

The *Callipyge* mutation site, situated in the proximal telomere region of chromosome 18, known as the *DLK1-GTL2* imprinted domain, is characterized by a remarkably preserved A-G mutation [20]. This domain spans approximately 20 kb [21]. Furthermore, mice and humans share similar homology regions on chromosomes 12 and 14 [22,23]. This area of study centers on a significant quantity of imprinted genes that have been characterized. The gene products of these imprinted genes primarily encompass growth hormones, cell cycle regulators, nutrient transport proteins, and diverse non-coding RNA molecules, all of which are essential for governing growth and development [24]. The genetic mechanism underlying the *Callipyge* gene is distinguished by its polar predominance. This suggests that in the heterozygous state, only the C allele is paternally transmitted, resulting in the manifestation of the *Callipyge* phenotype in sheep. Conversely, if heterozygous offspring inherit the C allele maternally or form a CC homozygous genotype, the sheep display a normal phenotype, precluding the manifestation of the *Callipyge* phenotype [25,26]. Multiple studies have documented the capacity of the Callipyge gene to not only induce hypertrophy in the rump muscles of sheep but also enhance the efficiency of slaughter, increase carcass weight, and augment the proportion of lean meat [27,28,29].

Because of their vital role, the polymorphisms of these genes were explored in the Tibetan sheep population and their impact on pre-weaning growth traits was examined. This study contributes to the evaluation of *MSTN* and *Callipyge* genes as potential markers for growth marker-assisted selection (MAS) in Tibetan sheep breeding, laying the groundwork for the incorporation of molecular markers in breeding programs.

## 2. Materials and Methods

### 2.1. Experimental Animals

The experimental group comprised 313 purebred, single-parity Tibetan sheep (male = 180, female = 133) born between January and February 2022 in Gangcha County, Haibei Tibetan Autonomous Prefecture. The sheep were raised on the Qinghai Provincial Sheep Breeding and Promotion Center ranch, where they were allowed to graze freely and had unlimited access to water and grass. Growth traits such as body weight (BW), body length (BL), body height (BH), chest circumference (ChC), chest depth (ChD), chest width (ChW), hip width (HW), and cannon circumference (CaC) were observed from birth until weaning at four months of age. The animal experiments adhered to the regulations outlined in the approved “Guidelines for animal care and use” manual by the Animal Care and Use Committee.

### 2.2. Sample Collection and Primer Design

Ear tissues were collected and preserved in a 75% alcohol solution, followed by DNA extraction using a DNA extraction kit from TIANGEN, Beijing, China. Custom-designed primers were utilized for all exons of the *MSTN* and *Callipyge* genes with the assistance of Primer3 v0.4.0 [30]. The *MSTN* gene consists of three exons, and the entire region of each exon was targeted by specific primers. Conversely, the *Callipyge* gene sequence was obtained from GenBank (AF401294), and a set of primers was developed to target this region. Additional details regarding the primers employed can be found in Table 1.

### 2.3. SNP Identification and Sequencing

We performed PCR reactions in a reaction volume of 30 μL, comprising 1.0 μL of DNA, 15 μL of 2×Taq PCR Master Mix, 1.0 μL of each primer, and double-distilled water (ddH_2_O) to reach the intended volume. We used a Bio-Rad S1000 thermal cycler (Bio-Rad, Hercules, CA, USA) for amplification, which consisted of an initial denaturation step at 94 °C for 2 min, 35 cycles of denaturation for 10 s at 94 °C, followed by annealing at 60 °C for 30 s, and elongation at 72 °C for 60 s. In the final step, PCR products were visualized on 1.0% agarose gel electrophoresis to determine the quality and quantity of the amplicons. Mutations were identified through Sanger sequencing using the Agilent 3730 system (Santa Clara, CA, USA). DNAMAN version 5.2.10 (Lynnon BioSoft, Vaudreuil, QC, Canada) was used for sequence analysis.

### 2.4. Population Genetic Index Calculation

Genetic parameters including allele frequency, heterozygosity (He), observed heterozygosity (Ho), effective allele numbers (Ne), and polymorphism information content (PIC) were evaluated utilizing Nei’s method [31]. The genotypes of the SNPs were examined for adherence to Hardy–Weinberg equilibrium (HW) in accordance with the Hardy–Weinberg law [32].

### 2.5. Statistical Analysis

The data analysis in this study was conducted using SPSS Statistics (Version 19, IBM SPSS Statistics, New York, NY, USA), with results presented as mean ± standard error. Associations between genotypes and individual growth traits were examined using general linear mixed models (GLMMs), with statistical significance defined as *p* < 0.05. The specific statistical model utilized in this research is detailed below:

Y = μ + Genotype + Sex + Interaction + ε

where Y represents the trait measured for each animal, including BW, BL, BH, ChW, ChD, ChW, HW, and CaC.

μ is the mean for the growth traits.

Genotypes represent the effects of the genotype.

Sex represents the effects of sex.

The interaction represents the interaction effect of sex and genotype.

ε is a random error, and it is assumed to be independent, with N (0, σ2) distribution.

## 3. Results

### 3.1. Polymorphism in Genes

**Figure 1:**
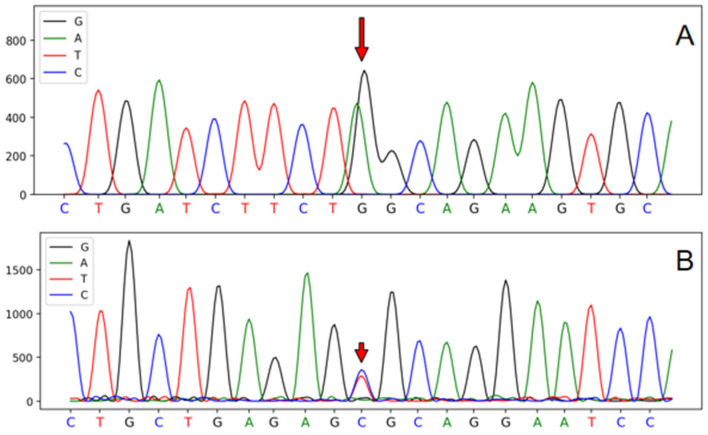
The peak map of mutated SNPs in *MSTN* and *Callipyge* of Tibetan sheep ^1^. ^1^ The sequences were subsequently analyzed utilizing the DNAMAN software. The identified location of the red arrow is the mutation site. (**A**) *MSTN* mutations marked by the red arrows are located in exon 2 of *MSTN* A592G (rs410961001). (**B**) SNP mutation C232T was identified in *Callipyge* (AF401294) at the sites marked with the red arrow.

An SNP locus was discovered within the *MSTN* and *Callipyge*, labeled A592G (rs410961001) in the *MSTN* and C232T in the *Callipyge* (AF401294). The sequencing peak maps for these genes illustrating the mutated sites are shown in Figure 1.

### 3.2. Population Genetic Analysis

*Ne* was calculated for each SNP and varied between 1 and 2. The allele frequencies of the SNPs were found to be in Hardy–Weinberg equilibrium. The PIC revealed that the SNP in *MSTN* was categorized as a locus with low-grade polymorphism (PIC ≤ 0.25), whereas the SNP in *Callipyge* was classified as a locus with moderate polymorphism (0.25 < PIC ≤ 0.5) (Table 2).

### 3.3. Association Analyses of Sex with Growth Traits

**Figure 2:**
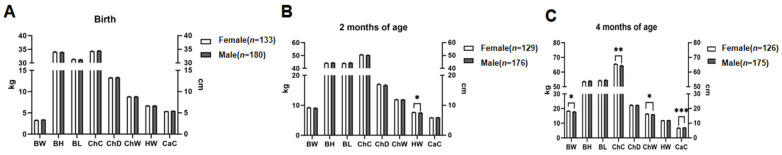
The association between sex and growth traits in Tibetan sheep ^1^. ^1^ (**A**) Analyses of the associations between sex and growth traits in Tibetan sheep at birth. (**B**) Analyses of the associations between sex and growth traits in Tibetan sheep at 2 months of age. (**C**) Analyses of the associations between sex and growth traits in Tibetan sheep at 4 months of age. BW stands for body weight; BL stands for body length; BH stands for body height; ChC stands for chest circumference; ChD stands for chest depth; ChW stands for chest width; HWstands for hip width; CaC stands for cannon circumference. Different superscript letters indicate significant differences at different levels of significance, with * representing *p* < 0.05, ** representing *p* < 0.01, and *** representing *p* < 0.001.

The results revealed a statistically significant relationship between sex and various growth traits at different ages, including HW at 2 months, BW and ChW at 4 months (*p* < 0.05), and ChC and CaC at 4 months (*p* < 0.01). Nevertheless, no significant associations were found between sex and other growth traits at birth, 2 months, or 4 months of age *(p* > 0.05; Figure 2).

### 3.4. Analyses of SNPs Associated with Growth Traits

**Figure 3:**
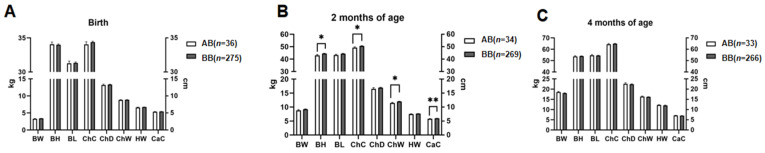
The association of SNPs in *MSTN* with Tibetan sheep’s growth traits ^1^. ^1^ (**A**) Association analysis of SNPs in *MSTN* and growth traits in Tibetan sheep at birth. (**B**) Association analysis of SNPs in *MSTN* and growth traits in Tibetan sheep at 2 months of age. (**C**) Association analysis of SNPs in *MSTN* and growth traits in Tibetan sheep at 4 months of age. AB represents the heterozygous mutant genotype; BB represents the homozygous mutant genotype; BW stands for body weight; BL stands for body length; BH stands for body height; ChC stands for chest circumference; ChD stands for chest depth; ChW stands for chest width; HWstands for hip width; CaC stands for cannon circumference. Different superscript letters indicate significant differences at different levels of significance, with * representing *p* < 0.05, ** representing *p* < 0.01.

**Figure 4:**
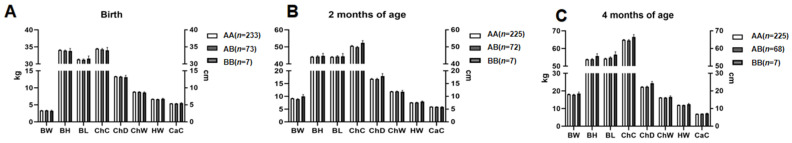
The association of SNPs in *Callipyge* with Tibetan sheep’s growth traits ^1^. ^1^ (**A**) Association analysis of SNPs in *Callipyge* and growth traits in Tibetan sheep at birth. (**B**) Association analysis of SNPs in *Callipyge* and growth traits in Tibetan sheep at 2 months of age. (**C**) Association analysis of SNPs in *Callipyge* and growth traits in Tibetan sheep at 4 months of age. AA represents the wild-type genotype; AB represents the heterozygous mutant genotype; BB represents the homozygous mutant genotype; BW stands for body weight; BL stands for body length; BH stands for body height; ChC stands for chest circumference; ChD stands for chest depth; ChW stands for chest width; HWstands for hip width; CaC stands for cannon circumference.

The analyses demonstrated statistically significant associations between the genotypes of *MSTN* and BL, ChW, and ChC (*p* < 0.05), and there was a highly significant association with CaC at 2 months of age (*p* < 0.01; Figure 3). However, all other associations between SNP in *MSTN* and *Callipyge* were not significant (*p* > 0.05; Figure 3 and Figure 4).

### 3.5. Analysis of Interaction Associations between Sex and SNPs with Growth Traits

The results revealed that there was a significant effect of sex on BW when the genotype of the *MSTN* gene was GA (*p* < 0.05). Additionally, when the genotype of *Callipyge* was CC, the effect of sex on CaC was highly significant at 2 months of age (*p* < 0.01, Table 3).

## 4. Discussion

Notably, *MSTN* and *Callipyge* are widely recognized as the most extensively studied genes that influence muscle cell movement and growth [33,34]. This study aimed to identify SNPs in two specific genes and establish their association with pre-weaning growth traits in Tibetan sheep. Furthermore, we aimed to identify effective loci that could enhance the growth performance of Tibetan sheep. The findings of this study will contribute to establishing a molecular foundation for improving the growth performance of Tibetan sheep.

Studies have indicated that sex is a significant determinant of the growth traits of domestic animals, with male animals typically exhibiting superior growth traits to their female counterparts [35]. In this study, it was found that the HW of ewes at 2 months of age and BW, ChC, and ChW at 4 months of age were significantly higher compared to rams. Furthermore, the CaC of rams at 4 months of age was observed to be greater than that of ewes. CaC is an indicator of somatic bone development, suggesting that the skeleton of rams is superior to that of ewes, specifically in terms of body size. It is crucial to acknowledge that the timeframe analyzed in this research aligns with the initial phases of sheep growth and maturation, potentially obscuring the observable reproductive benefits of rams [36].

The *MSTN* gene has been recognized as a suppressor of skeletal muscle growth and suggested as a potential gene for enhancing muscle production in sheep [37]. Importantly, mutations in *MSTN* that interfere with its expression have been associated with the phenomenon of “double-muscling” observed in various species. Prior research has demonstrated the identification of approximately 8–10 SNPs in this gene of sheep, as indicated in Table 4 [38,39,40,41,42,43,44], and these mutations lead to the production of nonfunctional proteins, ultimately promoting substantial muscle growth and increased muscularity [45]. Considering the significant importance of *MSTN* in animal breeding, using CRISPR/Cas9 technology to generate *MSTN*-edited brown sheep, the results revealed a substantial enhancement in myofiber diameter, mean daily gain, and overall body weight in *MSTN*-edited sheep [46,47]. In contrast, an analysis of variations within intron 1 of *MSTN* revealed no significant association with the average BW, weaning weight, or pre-weaning growth rate [48]. Additionally, g.6223G exhibited no discernible effect on the growth or weight of Australian white Suffolk, Bordeaux Set, or Lincoln sheep [44]. Our study found that the mutation site significantly affected the BH, ChC, Chw, and CaC in Tibetan sheep at 2 months of age. This observation indicated that the mutation site plays a crucial role in the early growth and development of Tibetan sheep, highlighting its potential as a molecular marker for evaluating early growth traits.

Conversely, only 2 SNPs have been identified in the *Callipyge* gene of sheep as shown in Table 4 [49,50,51]. The *Callipyge* phenotype has been documented in sheep populations, with subsequent research establishing that the A→G mutation situated 32.8 kb upstream of *GTL2* serves as the principal factor influencing this phenotype. However, it is important to acknowledge that the mutation frequency at this locus is notably low [49]. Notably, no instances of A→G mutations were detected in the *Callipyge* gene analysis conducted on Chinese and foreign sheep breeds [52]. This study was consistent with the results that identified a C→T mutation located 35 base pairs upstream of the mutation site responsible for the manifestation of the *Callipyge* phenotype. The findings of the study indicate a significant association between the C→T mutation located upstream of the mutation site of the double muscle hip gene and the occurrence of hind breech [50,51]. Moreover, a study indicated that the mutation in question did not have a substantial influence on the growth characteristics of Hornless Tawset, Suffolk, and Texel breeds. Nonetheless, significant discrepancies in growth traits were noted in the Tan sheep [50]. Studies analyzed the association between genotype and multiple growth traits in a cohort of 83 mature Oula sheep. Their findings suggested that the genotype of the locus under study did not yield statistically significant effects on BW, ChC, or ChD (*p* > 0.05). Nevertheless, heterozygous individuals tended to display higher values than homozygous individuals. In the present study, the genotype of the locus under investigation had no significant impact on growth performance at birth, 2 months of age, and 4 months of age [23]. This finding was consistent with previous studies where they observed that the growth rate of mutant *Callipyge* sheep before weaning was comparable to that of normal sheep. Furthermore, the phenotypic outcomes of the three genotypes did not exhibit any discernible trend, except for a significant difference in the CaC of rams when the genotype was heterozygous for CC, which was higher than that of ewes (*p* < 0.01) [53]. In summary, the SNP of the *Callipyge* gene did not exhibit any discernible effect on the pre-weaning growth performance of Tibetan sheep. However, additional studies are required to determine their potential effects on muscle development.

## 5. Conclusions

In conclusion, this study identified polymorphisms in two significant candidate genes in Tibetan sheep and assessed their impact on growth traits at birth and 2 and 4 months of age. Our findings reveal an association between the SNP locus of *MSTN* and growth and development at 2 months of age, suggesting its potential utility as a molecular marker for early growth traits in Tibetan sheep.

## Figures and Tables

**Table 1:** Primer information of *MSTN* and *Callipyge* genes in Tibetan sheep.

Primer Names	Primer Sequences (5′–3′)	Size (bp)	Tm (°C)
*Callipyge*	F:TGAAAACGTGAACCCAGAAGC	493	60
	R: GGCAGGAGAGACGGTTAAT		
*MSTN*-Exon1	F: ATCACAGATCCCGACGACAC	704	60
	R: CTCTTTGCCCTCCTCCTTAC		
*MSTN*-Exon2	F: CATAGATTGACATGGAGGCG	601	60
	R: TTTATTGGGTACAGGGCTAC		
*MSTN*-Exon3	F: CCATAAAGGCAGAATCAAGC	736	60
	R: TGTTGTGATGGTTAAATGCC		

**Table 2:** Population genetic analyses of *MSTN* and *Callipyge* in Tibetan sheep ^1^.

SNP	Gene Frequency		N	Ho	He	PIC	Ne	HW
	A	B						
*MSTN*	0.058	0.942	311	0.116	0.109	0.221	1.122	ns
*Callipyge*	0.861	0.139	312	0.234	0.240	0.404	1.316	ns

^1^ A represents the wild-type genotype, B represents the mutant genotype, N represents group size, Ho for homozygosity, He for heterozygosity, PIC for polymorphism information content, Ne for effective allele numbers, HW for Hardy–Weinberg equilibrium, and ns for non-significance.

**Table 3:** Interaction association analyses between sex and SNPs in *MSTN* and *Callipyge* with growth traits in Tibetan sheep ^1^.

State	SNP	Trait	Genotype	Female	Male	*p*-Value
Birth	*MSTN*	BW	AB	3.12 ± 0.10 ^b^	3.51 ± 0.13 ^a^	0.020
			BB	3.37 ± 0.05	3.40 ± 0.04	0.587
			*p*-value	0.044		
2 months	*Callipyge*	CaC	AA	5.88 ± 0.04 ^B^	6.04 ± 0.03 ^A^	0.003
			AB	5.99 ± 0.07	5.85 ± 0.06	0.123
			BB	6.03 ± 0.22	5.70 ± 0.19	0.249
			*p*-value	0.007		

^1^ AA represents the wild-type genotype; AB represents the heterozygous mutant genotype; BB represents the homozygous mutant genotype; BW stands for body weight; CaC stands for cannon circumference. Within a row, means denoted by different superscript letters are deemed to be significantly different at the 0.05 level of significance for ^a,b^ and at the 0.01 level of significance for ^A,B^.

**Table 4:** SNPs have been identified in the sheep *MSTN* and *Callipyge* genes.

Gene	Breed	SNP
*MSTN*	Dubo sheep, Tan sheep, small-tailed Han sheep	rs129059715
	Texel × Altai crossbred sheep	g.6723G>A
	East Friensian sheep	3′-UTR-272, 5-UTR-176
	Tan sheep	rs417816017
	Charollais sheep	g.2449G>C
	Nilagiri sheep	g.5622G>C
	New Zealand Romney sheep	g.6223G
*Callipyge*	Tausset sheep	A→G (at 211 bp in AF401294 amplified region)
	Tibetan sheep	C→T (at 176 bp in AF401294 amplified region)

## Data Availability

The data will be made available on request.
